# Effect of a plant sterol, fish oil and B vitamin combination on cardiovascular risk factors in hypercholesterolemic children and adolescents: a pilot study

**DOI:** 10.1186/1475-2891-12-7

**Published:** 2013-01-08

**Authors:** Iveta Garaiova, Jana Muchová, Zuzana Nagyová, Csilla Mišľanová, Stanislav Oravec, Andrej Dukát, Duolao Wang, Sue F Plummer, Zdeňka Ďuračková

**Affiliations:** 1 Research Department, Obsidian Research Ltd, Port Talbot, UK; 2 Institute of Medical Chemistry, Biochemistry and Clinical Biochemistry, Faculty of Medicine, Comenius University, Bratislava, 813 72, Slovakia; 3 Juvenalia Paediatric Centre, Dunajská Streda, Slovakia; 4 Department of Bioactive Substances and Nutritional Screening, Slovak Medical University, Bratislava, Slovakia; 5 2nd Department of Internal Medicine, Faculty of Medicine, Comenius University, Bratislava, Slovakia; 6 Department of Medical Statistics, London School of Hygiene and Tropical Medicine, London, UK

**Keywords:** Cholesterol, Plant sterols, Fish oil, B vitamins, Children, Adolescents

## Abstract

**Background:**

Assessment of cardiovascular disease (CVD) risk factors can predict clinical manifestations of atherosclerosis in adulthood. In this pilot study with hypercholesterolemic children and adolescents, we investigated the effects of a combination of plant sterols, fish oil and B vitamins on the levels of four independent risk factors for CVD; LDL-cholesterol, triacylglycerols, C-reactive protein and homocysteine.

**Methods:**

Twenty five participants (mean age 16 y, BMI 23 kg/m^2^) received daily for a period of 16 weeks an emulsified preparation comprising plant sterols esters (1300 mg), fish oil (providing 1000 mg eicosapentaenoic acid (EPA) plus docosahexaenoic acid (DHA)) and vitamins B12 (50 μg), B6 (2.5 mg), folic acid (800 μg) and coenzyme Q10 (3 mg). Atherogenic and inflammatory risk factors, plasma lipophilic vitamins, provitamins and fatty acids were measured at baseline, week 8 and 16.

**Results:**

The serum total cholesterol, LDL- cholesterol, VLDL-cholesterol, subfractions LDL-2, IDL-1, IDL-2 and plasma homocysteine levels were significantly reduced at the end of the intervention period (*p*<0.05). The triacylglycerols levels decreased by 17.6%, but did not reach significance. No significant changes in high sensitivity C-reactive protein, HDL-cholesterol and apolipoprotein A-1 were observed during the study period. After standardisation for LDL cholesterol, there were no significant changes in the levels of plasma γ-tocopherol, β-carotene and retinol, except for reduction in α-tocopherol levels. The plasma levels of n-3 fatty acids increased significantly with the dietary supplementation (*p*<0.05).

**Conclusions:**

Daily intake of a combination of plant sterols, fish oil and B vitamins may modulate the lipid profile of hypercholesterolemic children and adolescents.

**Trial registration:**

Current Controlled Trials ISRCTN89549017

## Background

Atherosclerotic cardiovascular disease is the number one cause of death worldwide with over 2.0 million deaths in the European Union and an estimated total cost of €192 billion a year [1]. Atherosclerosis can begin in childhood with the development of arterial fatty streaks. Assessment of the independent risk factors for CVD, particularly low density lipoprotein (LDL) cholesterol in children and adolescents, can predict clinical manifestations of atherosclerosis in adulthood and therefore there is a growing demand for lipid screening in childhood [2,3].

Improvements in diet and lifestyle can reduce CVD risk factors, but failure to respond to such interventions can result in pharmacological intervention in children from the age of 8 years with the use of statins recommended for LDL-cholesterol levels ≥190 mg/dl (≥4.9 mmol/l) or when LDL-cholesterol ≥160 mg/dl (≥4.1 mmol/l) and additional risk factors are present [3,4].

Plant sterols/stanols and n-3 long-chain polyunsaturated fatty acids (PUFA) are increasingly being used for the prevention and management of hypercholesterolemia, hyperlipidemia and other risk factors such as inflammation and thrombosis. The intakes of plant sterol/stanol esters can reduce blood LDL-cholesterol levels in children with familial hypercholesterolemia [5-7]. The European Food Safety Authority (EFSA) has approved the daily intake of 1.5 – 2.4 g of plant sterol/stanol esters to achieve a blood LDL-cholesterol reduction of 7-10.5% in 2-3 weeks in an adult population [8].

The cardioprotective effects of n-3 PUFA appear to be due a multifaceted synergistic process involving reduction of triacylglycerols (TAG), very-low-density lipoprotein (VLDL), increasing levels of antiinflammation together with regulation of transcription factors and gene expression, membrane fluidity and antiarrhythmic and antithrombotic effects [9]. In children with hyperlipidemia, supplementation with DHA has been found to be associated with a significant shift in lipoprotein subclasses towards large, buoyant LDL particles and restoration of brachial artery endothelial function [10,11].

Reduction of total homocysteine levels by folic acid, vitamin B6 and vitamin B12 can contribute to the prevention of CVD [12] and folic acid supplementation has been found to reduce total homocysteine and high sensitivity C-reactive protein (hsCRP) in children with familial hypercholesterolemia [13].

Therefore, the combination of these natural compounds may offer more comprehensive approach in the early management of CVD risk factors.

This pilot study investigates the impact of a combination of plant sterols, fish oil and B vitamins on the levels of four independent risk factors for CVD (LDL-cholesterol, TAG, hsCRP and homocysteine) in a group of hypercholesterolemic children and adolescents.

## Methods

### Subjects

Thirty children and adolescents, registered with Juvenalia Pediatric Centre, Dunajska Streda, Slovakia, were invited to participate in the study based on the universal screening of cholesterol and glycaemia in children aged 11 and adolescents aged 17 according to Ministry of Health guidelines MZ13010/2004. Written informed consent was obtained from the parents or guardians before participating in the study. Subjects with initial total cholesterol levels ≥ 4.1 mmol/l (≥159 mg/dl) [14] despite completing a 3 month period of assigned dietary lifestyle change were selected for inclusion in this study. Genetic diagnosis of familial hypercholesterolemia or familial combined hyperlipidemia had not been determined at this stage of investigation. None of the subjects had received lipid or cholesterol lowering drugs, were diabetic, hypertensive or had a history of cardiovascular diseases.

### Study design and dietary assessment

Ethical Approval was granted by the Medical School Ethics Board, Comenius University in Bratislava, Slovakia (2007). During the 16 week study period, participants received daily two 5 ml doses of an emulsified preparation of plant sterols esters (1300 mg) and fish oil (providing 590 mg EPA and 410 mg DHA) together with vitamins B12 (50 μg), and B6 (2.5 mg), folic acid (800 μg) and coenzyme Q10 (3 mg) supplied by Cultech Ltd., Port Talbot, United Kingdom. The fatty acid composition of the intervention was as follows: Myristic acid (14:0) 4.84%, Palmitic acid (16:0) 10.76%, Palmitoleic acid (16:1) 3.67%, Stearic acid (18:0) 4.05%, Oleic acid (18:1, n-9) 1.7%, Linoleic acid (18:2, n-6) 10.05%, Arachidonic acid (20:4, n-6) 1.8%, EPA (20:5, n-3) 24.08%, DHA (22:6, n-3) 16.32%, Others 24.73%.

Compliance to product assessed from the weekly records of daily intervention intake was 88.2%.

All subjects were instructed to maintain their usual dietary and exercise patterns throughout the study period and to monitor any changes during the study, parents/guardians were asked to complete a participant diet habit and physical activity pattern questionnaire at baseline and upon completion of the study. The questionnaire consisted of 4 questions on meal and drink patterns, 9 questions on the intake of various foods that are a common part of a typical diet in the country and 3 questions relating to physical activities. Data on food intake and physical activity were expressed as frequencies ranging from several times per day to never. The questionnaires were assessed using a scoring system and summary scores at baseline and upon completion of the study were compared.

### Compliance

Of the thirty subjects, five withdrew from the study due to product palatability or nausea. Twenty five subjects therefore completed the intervention.

### Clinical and anthropometric assessment

Resting blood pressure levels were measured in the left arm in the sitting position by sphygmomanometer (Omron M3, Intellisense, Japan). Body weight and height were measured without shoes and with light clothing using a digital weighing and measuring station with automatic body mass index (BMI) calculation (kg/m^2^, SECA 764, Germany). Overweight was defined as BMI ≥ 90 percentile on the age- and gender-specific BMI for age growth chart and obese as BMI ≥ 97 percentile. Waist circumferences were measured in cm with an inelastic tape to the nearest mm, with the subject in a standing position.

### Biochemical analysis

Venous blood samples were collected after 12-hours overnight fast. Within 1 h of collection, blood was centrifuged (1200 x g, 10 min), serum and plasma (EDTA as an anticoagulant) were obtained and frozen at -80°C until analysis.

The fasting serum analysis of total cholesterol, TAG, hsCRP, apolipoprotein A-1 and glucose was determined at an accredited Clinical biochemistry and haematology laboratory using a Hitachi 911 Analyzer (Roche Diagnostics, Switzerland). Lipoproteins were determined by Lipoprint^®^ LDL system (Quantimetrix Corp. CA, USA) according to manufacturer’s instructions. The atherogenic lipoprotein profile is characterised by the presence of VLDL, IDL-1 (intermediate density), IDL-2 and small dense LDL-3 to LDL-7 subfractions [15].

Plasma homocysteine, methionine and cysteine were determined on a HPLC system [16,17]. The internal standard (penicillamine) and sodium borohydride (1 mol/l in 0.1 mol/l NaOH) were added to 100 μl of plasma, mixed and incubated at 50°C for 30 minutes. Perchloric acid in EDTA (0.6 mol/l in 1 mmol/l) was added and samples centrifuged at 14,000 rpm for 5 minutes. The supernatant was diluted 1:1 ratio into the mobile phase (50 mmol/l sodium phosphate, 1.0 mmol/l ion-pairing reagent 1-octanesulfonic acid and 6% acetonitrile (v/v), adjusted to pH 2.7 with 85% phosphoric acid) and injected (50 μl) onto a Purospher^®^ STAR RP-18e column (250x4.6 mm I.D., 5 μm, Merck) at a flow rate of 0.8 ml/min at ambient temperature with a Coulochem II detector (ESA, USA) equipped with an analytical cell (model 5010A) and a guard cell (model 5020).

Plasma α-tocopherol, γ-tocopherol, β-carotene and retinol were determined by a reversed phase HPLC method [18] using a Nucleosil 120-5 C18 column (250x4.6 mm, 5 μm I.D., protected by a Nucleosil C18 precolumn, Watrex, Czech Republic) with fluorescent detector (0-4 min at 330/470 excitation/emission; 4.1-20 min T 298/328) and detector in the visible range at 450 nm (Watrex, Czech Republic). Flow injection was 20 μl. The mobile phase consisted of acetonitrile:tetrahydrofuran:methanol containing BHT:1% ammonium acetate (67.4 : 22 : 6.8 : 3.8, v/v/v/v). Flow rate was 1.2 ml/min and column oven was set at 29°C.

Fatty acid methyl esters (FAME) were prepared using an adaptation of a method previously reported [19]. The extracted lipid sample (75 μl) was incubated with 2.5% sulphuric acid in dry methanol: toluene (2:1 v/v) for 2 h at 70°C. FAME were extracted using hexane, dried down under nitrogen and resuspended in isooctane. The FAME were analysed by gas chromatography using an Agilent Technologies 6890 N gas chromatograph, fitted with a 7683 series injector and an Elite-225 column (30 m x 0.25 mm I.D., 0.25 μm film thickness; Perkin Elmer, USA). The column temperature was 170°C for 3 min to 220°C at 4°C/min, hydrogen as carrier gas (1.1 ml/min). The FAME were identified by comparison of their retention times with standards (Sigma, Dorset, UK) and nonadecanoic acid (C19:0) was used as an internal standard.

The intraassay coefficient of variation (CV) for measured parameters ranged between 0.8 to 6.8% and the interassay CV range was between 1.2 to 10.5%.

### Statistical analysis

Data were analysed by a mixed model, taking account of repeated measurements. In this model, the visit (week=0, 8 and 16) was treated as a fixed effect whereas the subject was treated as a random effect. During the study, some subjects dropped out, resulting in some incomplete observations which were not imputed but were assumed to be missing at random in the mixed model analysis. The estimated differences between week 8, 16 and baseline were derived from the mixed model and were reported together with their 95% confidence intervals. Reported *P*-values are two-sided and a *P*-value of <0.05 was considered statistically significant, and all statistical analyses were carried out by using the Statistical Analysis System (SAS) version 9.2 (SAS Institute, Inc., Cary, NC, USA).

## Results

The baseline characteristics for the total population and the screening age groups are shown in Table 1. In addition, baseline characteristics of the subjects by gender are presented in Additional file 1: Table S1. Among the subjects, 16/25 girls (81.2% menstruating) and 9/25 boys, 20% (5 boys) were considered overweight and 12% (2 boys/1 girl) obese. 12 subjects had waist circumference and 11 had blood pressure levels above the reference range [14]; fasting glucose levels were acceptable (range: 3.8 - 5.8 mmol/l).

**Table 1:** **Baseline characteristics of subjects**^**a**^

**Parameter**	**All**	**10-16 years old**	**≥ 17 years old**
	**(n=25)**	**(n=10)**	**(n=15)**
Age (years)	16.37 (3.75)	11.56 (1.34)	18.43 (1.08)
Weight (kg)	63.66 (23.49)	51.60 (19.00)	71.70 (23.26)
BMI (kg/m^2^)	22.99 (6.10)	21.10 (6.10)	24.26 (5.97)
Overweight (n)	5/25	2/10	3/15
Obese (n)	3/25	1/10	2/15
Waist circumference (cm)	85.18 (15.58)	80.55 (13.89)	88.27 (16.33)
Fasting Glucose (mmol/l)	4.97 (0.48)	5.01 (0.29)	4.94 (0.59)
Blood Pressure			
Systolic (mmHg)	122.60 (13.78)	120.00 (7.82)	124.33 (16.68)
Diastolic (mmHg)	78.80 (9.38)	79.00 (6.99)	78.67 (10.93)

^a^Data presented as mean (standard deviation); n- number of subjects per group.

After 16 weeks, systolic blood pressure and glucose levels decreased significantly by 3.59% (*P*=0.0457) and 4.02% (*P* =0.0275), respectively with no change in the diastolic blood pressure. BMI increased significantly (*P*=0.0498) only in adolescents aged 17 and over.

### Atherogenic and inflammatory risk markers

Significant reductions in Total cholesterol (7.74%), LDL-cholesterol (8.45%) and VLDL-cholesterol (19%) were observed at 16 weeks with no change in HDL-cholesterol, apolipoprotein A1 or hsCRP levels (Table 2). TAG levels decreased over 16 weeks by 17.6%, but did not reach significance. Homocysteine and methionine levels were significantly lower at week 16 (*P*<0.0001 for both), whilst cysteine levels increased (*P=*0.0002).

**Table 2:** Serum lipids and other atherogenic and inflammatory risk markers

**Parameter**	**Baseline mean (SD)**	**Change at week 8**	**95% CI**	***P*-value**	**Change at week 16**	**95% CI**	***P*-value**
Total-C (mg/dl)^a^	205.56 (44.10)	-5.96	-15.19 to 3.27	0.2005	-15.92	-25.15 to -6.69	0.0011
LDL-C (mg/dl)^a^	124.52 (38.20)	-2.96	-9.84 to 3.92	0.3912	-10.52	-17.40 to -3.64	0.0035
HDL-C (mg/dl)^a^	52.04 (10.83)	-1.96	-5.48 to 1.56	0.2683	0.32	-3.20 to 3.84	0.8557
Atherogenic Index	4.08 (1.08)	0.02	-0.25 to 0.28	0.8940	-0.36	-0.62 to -0.09	0.0095
TAG (mmol/l)	1.42 (0.66)	0.22	-0.17 to 0.61	0.2586	-0.24	-0.63 to 0.15	0.2191
Apolipoprotein A1 (g/l)	1.50 (0.25)	0.05	-0.04 to 0.14	0.3073	0.02	-0.07 to 0.11	0.7086
hsCRP (mg/l)	1.86 (2.03)	-0.11	-1.05 to -0.83	0.8159	0.86	0.08 to 1.81	0.0729
Homocysteine (μmol/l)	8.51 (3.14)	-2.88	-3.61 to -2.14	<0.0001	-3.25	-3.98 to -2.51	<0.0001
Methionine (μmol/l)	28.55 (4.32)	-5.13	-7.14 to -3.13	<0.0001	-8.58	-10.59 to -6.57	<0.0001
Cysteine (μmol/l)	173.87 (33.25)	44.00	25.98 to 62.03	<0.0001	36.37	18.34 to 54.39	0.0002
*Lipoprotein Subfractions (mg/dl)*^*a*^							
VLDL	28.84 (6.34)	-1.08	-3.86 to 1.70	0.4378	-5.48	-8.26 to -2.70	0.0002
IDL-1	28.48 (10.02)	-1.88	-4.40 to 0.64	0.1399	-5.20	-7.72 to -2.68	0.0001
IDL-2	11.12 (4.21)	-0.12	-1.25 to 1.01	0.8322	-2.08	-3.21 to -0.95	0.0006
IDL-3	16.40 (7.71)	3.04	0.31 to 5.77	0.0297	-1.08	-3.81 to 1.65	0.4299
LDL-1	47.60 (14.78)	0.48	-3.80 to 4.76	0.8226	1.28	-3.00 to 5.56	0.5505
LDL-2	19.60 (13.44)	-4.68	-7.84 to -1.52	0.0045	-3.44	-6.60 to -0.28	0.0335
LDL-3	1.32 (1.93)	-0.08	-1.02 to 0.86	0.8642	0.00	-0.94 to 0.94	1.0000

Data are presented as mean (standard deviation);

C - cholesterol; LDL - low density lipoprotein; HDL - high density lipoprotein; Atherogenic Index – Total cholesterol:HDL cholesterol; TAG – triacylglycerols; hsCRP- high sensitivity C-reactive protein; VLDL - very low density lipoprotein; IDL - intermediate density lipoprotein,

^a^To change to mmol/l, multiply the value with 0.02586.

*P*-value derived with mixed model.

Analysis of cholesterol subfractions showed significant reduction in IDL-1, IDL-2 and LDL-2 levels at week 16 compared to baseline (Table 2). Lipoprint^®^ profiles for all participants are presented in Additional file 2: Table S2.

Subgroup analysis on the basis of age indicates a significant reduction in the Total cholesterol, LDL-cholesterol and LDL-2 subfraction in adolescents over 17 years old at week 16 (*P=*0.0049, *P=*0.0176 and *P* =0.0264, respectively), but only a trend towards significance in the younger age group (*P=*0.0783 for Total cholesterol and *P=*0.0833 for LDL-cholesterol). Significant reductions were recorded after 16 weeks in both age groups for VLDL-cholesterol and homocysteine levels (*P* for all <0.05). In addition, there was a marked reduction in the TAG levels, which reached significance for the 10-16 year old children (*P*=0.0355). No significant changes in hsCRP, HDL-cholesterol and apolipoprotein A1 were observed in either age group.

### PUFA profile

Plasma levels of EPA and DHA increased significantly over the study period by 220% and 130% (Table 3), whereas the levels of arachidonic acid (AA) and dihomo-γ-linolenic acid (DGLA) decreased significantly by 18% and 21%, respectively.

**Table 3:** Polyunsaturated fatty acids profile in plasma

**Parameter (μg FA/ml)**	**Baseline mean (SD)**	**Change at week 8**	**95% CI**	***P*-value**	**Change at week 16**	**95% CI**	***P*-value**
ALA	13.50 (8.50)	-2.08	-4.71 to 0.56	0.1194	-3.71	-6.35 to -1.08	0.0067
EPA	8.73 (4.09)	17.96	12.42 to 23.50	<0.0001	10.47	4.93 to 16.00	0.0004
DPA	8.24 (2.88)	3.63	2.10 to 5.16	<0.0001	0.56	-0.97 to 2.09	0.4665
DHA	32.91 (8.24)	18.39	12.05 to 24.73	<0.0001	9.93	3.60 to 16.27	0.0028
EPA+DHA	41.63 (10.68)	36.35	25.16 to 47.55	<0.0001	20.40	9.21 to 31.6	0.0006
LA	624.25 (137.45)	7.30	-52.75 to 67.34	0.8081	-55.81	-115.86 to 4.24	0.0678
GLA	10.01 (4.40)	0.75	-1.83 to 3.32	0.5615	-1.55	-4.12 to 1.03	0.2330
DGLA	35.07 (13.03)	-3.32	-7.89 to 1.25	0.1550	-7.50	-12.07 to -2.93	0.0018
AA	148.24 (38.47)	-11.57	-27.37 to 4.24	0.1477	-26.56	-42.37 to -10.76	0.0015

Data are presented as mean (standard deviation);

FA- fatty acid; ALA – α-linolenic acid, 18:3 (n-3); EPA – eicosapentaenoic acid, 20:5 (n-3); DPA – docosapentaenoic acid, 22:5 (n-3); DHA – docosahexaenoic acid, 22:6 (n-3); LA – linoleic acid, 18:2 (n-6); GLA – γ-linolenic acid, 18:3 (n-6); DGLA – dihomo-γ-linolenic acid, 20:3 (n-6); AA – arachidonic acid, 20:4 (n-6); *P*-value derived with mixed model.

### Vitamins and provitamins

Reductions in plasma α-tocopherol (19.2%) and retinol (19.5%) levels were observed at the end of the intervention period with no changes in the levels of γ-tocopherol and β-carotene (Table 4). After standardisation for LDL cholesterol, only significant reduction was detected for α-tocopherol (*P*=0.0210).

**Table 4:** Lipophilic vitamins and provitamins

**Parameter**	**Baseline mean (SD)**	**Change at week 8**	**95% CI**	***P*-value**	**Change at week 16**	**95% CI**	***P*-value**
α-Tocopherol (mg/l)	13.09 (3.49)	-0.40	-1.65 to 0.85	0.5231	-2.52	-3.76 to -1.27	0.0002
α-Tocopherol/LDL-C^a^	10.79 (3.38)	-0.09	-1.26 to 1.08	0.8808	-1.39	-2.56 to -0.22	0.0210
γ-Tocopherol (mg/l)	0.69 (0.39)	0.51	0.27 to 0.76	0.0001	0.17	-0.08 to 0.42	0.1747
γ-Tocopherol/LDL-C^a^	0.56 (0.37)	0.48	0.26 to 0.69	0.0001	0.18	-0.03 to 0.40	0.0938
Retinol (mg/l)	0.87 (0.28)	-0.03	-0.13 to 0.07	0.5283	-0.17	-0.27 to -0.07	0.0015
Retinol/LDL-C^a^	0.71 (0.25)	0.03	-0.08 to 0.13	0.6230	-0.07	-0.17 to 0.04	0.2099
β-Carotene (mg/l)	0.81 (0.91)	-0.15	-0.43 to 0.12	0.2712	0.26	-0.53 to 0.02	0.0640
β-Carotene/LDL-C^a^	0.63 (0.76)	-0.11	-0.35 to 0.13	0.3708	-0.19	-0.43 to 0.05	0.1200

Data are presented as mean (standard deviation); ^a^Results multiplied by 1000.

*P*-value derived with mixed model.

### Background diet

No significant differences in dietary habits and physical activity patterns were observed over the study period (*P*=0.669, F=0.188), indicating that the changes in lipid profiles were unlikely to be the result of dietary alteration.

## Discussion

The present study shows that the combination of plant sterol esters (1300 mg), fish oil (1000 mg EPA+DHA) and B vitamins impacts on LDL-cholesterol and homocysteine levels in hypercholesterolemic children and adolescents. To our knowledge, this is the first report of the use of the combination of these natural compounds for early dietary management of potential CVD risk.

Elevated LDL-cholesterol level is a well-established independent risk factor for CVD. Reductions in the LDL-cholesterol levels by 8.7% in the group of 15 adolescents aged over 17 years and by 9.5% in the group of 10 children aged 10 to 16 years were observed. The results are in line with previous studies on the use of plant sterols and stanols in children and adolescents with hypercholesterolemia with the significant LDL-cholesterol reduction observed in range of 9 to 15% with daily intake ranging from 1.2 to 3 g of plant sterol or stanols [5-7,20,21]. It has been proposed that the mechanism of such cholesterol lowering involves competition between intestinal plant sterols/stanols and intestinal cholesterol for absorption in mixed micelles and upregulation of the enterocyte ATP-binding cassette transport proteins [22].

The atherogenic index (Total cholesterol to HDL-cholesterol ratio) improved during the study with no significant change in HDL-cholesterol, apolipoprotein A-1 and hsCRP.

Measurement of LDL subfractions may provide additional assessment of CVD risk. There is growing evidence that small dense LDL subfractions may be more atherogenic than larger less dense LDL subfractions due to their lower binding affinity for the LDL receptor, impaired clearance from the circulation, susceptibility to oxidative modification and easier penetration into the subendothelial space of the arterial wall, leading more readily to the formation of cholesterol deposits and inflammation [23]. In the present study, the combined effect of plant sterol esters, n-3 PUFA and B vitamins resulted in less atherogenic profile. The levels of the atherogenic lipoprotein VLDL, IDL-1 and IDL-2 subfractions decreased significantly in both age groups. However, only trace levels of the LDL-3 subfraction were found in 15 participants (2 in the age group 10-16 years) with no LDL-4 to LDL-7 subfractions detected. In addition, significant reductions in the predominant large dense LDL-2 subfraction was observed in both age groups without any change in non-atherogenic subfractions IDL-3 and LDL-1. In contrast to our results, Engler et al. [11] reported significantly increased LDL-1 subfraction by 91% and decreased small dense LDL-3 by 48% after supplementation with DHA (1.2 g/day) for 6 weeks in children with either initial LDL-cholesterol levels above 130 mg/dl (familial hypercholesterolemia) or LDL-cholesterol levels above 130 mg/dl or TAG >150 mg/dl or both (familial combined hyperlipidemia). To our knowledge there are no reports on the administration of plant sterols/stanols linked with LDL subfraction analysis in children and adolescents. In hypercholesterolemic adults, following plant sterol and stanols supplementation in various forms and doses, the results are inconsistent with no significant changes in LDL particle size [24] or a significant reduction in all LDL subfractions [25] or small LDL subfraction [26].

Accumulating evidence suggests that elevated plasma TAG may pose another significant independent risk for CVD [27]. The n-3 PUFA lowering effect on plasma TAG levels is well established in adults, but no significant changes in TAG levels in children and adolescents with dyslipidemia were seen after daily supplementation with 1.2 g of DHA [10,11]. In our study, the TAG levels decreased in both age groups, but a significant reduction was observed in the group of children aged 10 to 16 (baseline mean TAG level 1.1 mmol/l). Recently, a pooled analysis of 12 randomised controlled trials showed that plant sterols alone modestly lower TAG levels (by 6% or 0.12 mmol/l) in hypercholesterolaemic adults depending on baseline concentration [28]. Moreover, Micallef et al [29] showed a 22.6% reduction in overall cardiovascular risk of hyperlipidemic adults consuming the combination of 2 g/day plant sterols and 1.4 g/day omega 3 PUFA compared to 15.1% and 15.3% CVD risk reduction in the plant sterols and fish oil groups alone.

A reduction in lipid levels affects plasma levels of fat soluble vitamins [30,31]. In the present study, the concentrations of vitamins and provitamins were unchanged after standardisation for LDL-cholesterol levels, except for a reduction in α- tocopherol levels.

Elevated homocysteine concentration is an independent risk factor for ischemic stroke and thromboembolism in children [32,33] and is strongly influenced by folate and B vitamins status [34]. The levels of total homocysteine seen in the present study were within the normal ranges for both age groups agreeing with previous studies on healthy or hypercholesterolemic children [13,34-36]. However, we noted a significant reduction in total homocysteine by 3.25 μmol/l after 16 weeks intervention, similar to significant reduction by 3.99 μmol/l in children with familial hypercholesterolemia supplemented daily with 5 mg folate for 3 months [13]. A meta-analysis of observational studies in adults suggested that lowering total homocysteine levels by 3 μmol/l would reduce the risk of ischemic heart disease by 11% and stroke by 19% [37]. Results for the metabolites of the homocysteine metabolic pathway, methionine and cysteine, indicate that the intervention may have had an effect on the degradation of homocysteine through the transsulfuration pathway to cysteine rather than on re-methylation of homocysteine to methionine. Cysteine may be utilised in protein synthesis or as a precursor in the synthesis of glutathione.

The pre-emulsification of fish oil significantly improves absorption of EPA and DHA [19]. The addition of fish oil (590 mg EPA+410 mg DHA/day) into the emulsified intervention resulted in increased levels in plasma EPA by 220% and DHA by 130% and significant reduction of the n-6 PUFAs; AA and DGLA. Engler et al [38] reported increases in the DHA concentration and reduction in the n-6 PUFA in the plasma phospholipids of hyperlipidemic children after algal DHA oil supplementation (1.2 g/day). Reducing the n-6:n-3 fatty acid ratio in the diet may contribute to cardioprotective health in children and adolescents.

Our pilot study has limitations; this was an investigational uncontrolled study and the observed within-group differences may be subject to confounding factors. Also the small number of participants included in this study means that the study was underpowered to detect all statistical significant differences which could explain the observed trends for some results especially in the younger group (10-16 years old) with only 10 participants.

## Conclusions

An emulsified combination of plant sterols, fish oil and B vitamins has beneficial effects on recognised cardiovascular risk factors in hypercholesterolemic children/adolescents over a short term period following unsuccessful attempts to alter the profiles using only dietary means. These results are encouraging, but need to be confirmed with a large randomised placebo controlled study.

## Abbreviations

ALA: α-linolenic acid; AA: Arachidonic acid; BMI: Body mass index; CVD: Cardiovascular disease; DGLA: Dihomo-γ-linolenic acid; DHA: Docosahexaenoic acid; DPA: Docosapentaenoic acid; EDTA: Ethylenediaminetetraacetic acid; EFSA: European Food Safety Authority; EPA: Eicosapentaenoic acid; FA: Fatty acid; FAME: Fatty acid methyl esters; GLA: γ-linolenic acid; HDL: High- density lipoprotein; hsCRP: High sensitivity C-reactive protein; IDL: Intermediate density lipoprotein; LA: Linoleic acid; LDL: Low-density lipoprotein; PUFA: Polyunsaturated fatty acids; TAG: Triacylglycerols; VLDL: Very-low-density lipoprotein.

## Competing interests

Sue F Plummer is Technical Director of Cultech Ltd. All other authors declared that they have no competing interests.

## Authors’ contributions

IG and JM contributed to study design, performance of biochemical assays, collection and interpretation of data and manuscript writing; ZN was responsible for recruitment and clinical and anthropometric assessment; CM, SO and AD were responsible for performing the biochemical assays and interpretation of data; DW was responsible for statistical analysis of data; SFP and ZĎ contributed to study design, procurement of the funding, Ethical Committee documents, coordination of the study and interpretation of data. All authors assisted in reviewing/editing the manuscript and approved the final manuscript.

## Supplementary Material

Additional file 1 **Table S1.** Baseline characteristics of the subjects by gender. Description of data: The file contains a detailed breakdown of the baseline characteristics by gender. Click here for file

Additional file 2 **Table S2.** Lipoprint^®^ profiles for all subjects. Description of data: The file contains detailed information on the lipoprotein subfractions for each subject. Click here for file
