# Supplementary material for: Effect of a plant sterol, fish oil and B vitamin combination on cardiovascular risk factors in hypercholesterolemic children and adolescents: a pilot study
Source: Nutr J. 2013 Jan 8;12:7. doi: 10.1186/1475-2891-12-7 (PMC3549748; doi:10.1186/1475-2891-12-7)
Supplement: Additional file 1 — Table S1. Baseline characteristics of the subjects by gender. Description of data: The file contains a detailed breakdown of the baseline characteristics by gender. [file 1475-2891-12-7-S1.docx]

## Table S1 Baseline characteristics of subjects by gender^a^

| **Parameter** | **All (n)**  **(B/G =9/16)** | **10-16 years old (n)**  **(B/G =3/7)** | **≥ 17 years old (n)**  **(B/G=6/9)** |
| --- | --- | --- | --- |
| *Weight (kg)*  Boys  Girls | 79.06 (28.47)  55.00 (15.11) | 50.67 (13.65)  52.00 (21.88) | 93.25 (22.29)  57.33 (7.45) |
| *BMI (kg/m^2^)*  Boys  Girls | 26.58 (6.50)  20.98 (5.00) | 21.27 (3.52)  21.03 (7.18) | 29.24 (6.10)  20.94 (2.86) |
| *Overweight (n)*  Boys  Girls | 5  0 | 2  0 | 3  0 |
| *Obese (n)*  Boys  Girls | 2  1 | 0  1 | 2  0 |
| *Waist circumference (cm)*  Boys  Girls | 95.56 (17.15)  79.34 (11.42) | 84.00 (12.49)  79.07 (15.12) | 101.33 (16.98)  79.56 (8.55) |
| *Fasting Glucose (mmol/l)*  Boys  Girls | 5.35 (0.47)  4.76 (0.35) | 5.18 (0.20)  4.94 (0.31) | 5.43 (0.56)  4.61 (0.32) |
| *Blood Pressure*  *Systolic (mmHg)*  Boys  Girls  *Diastolic (mmHg)*  Boys  Girls | 128.89 (17.46)  119.06 (10.20)    82.22 (8.70)  76.88 (9.46) | 113.33 (11.55)  122.86 (3.93)    75.00 (5.00)  80.71 (7.32) | 136.67 (14.72)  116.11 (12.69)    85.83 (8.01)  73.89 (10.24) |

^a^Data presented as mean (standard deviation); n- number of subjects per group; B-boys;

G-girls
