# Supplementary material for: Effect of a plant sterol, fish oil and B vitamin combination on cardiovascular risk factors in hypercholesterolemic children and adolescents: a pilot study
Source: Nutr J. 2013 Jan 8;12:7. doi: 10.1186/1475-2891-12-7 (PMC3549748; doi:10.1186/1475-2891-12-7)
Supplement: Additional file 2 — Table S2. Lipoprint® profiles for all subjects. Description of data: The file contains detailed information on the lipoprotein subfractions for each subject. [file 1475-2891-12-7-S2.docx]

## Table S2 Lipoprint® profiles for all subjects

|  | | | **Lipoprotein Subfractions (mg/dl)** | | | | | | | |
| --- | --- | --- | --- | --- | --- | --- | --- | --- | --- | --- |
| **Participants** | **Boy/Girl** | **Week** | **VLDL** | **IDL-1** | **IDL-2** | **IDL-3** | **LDL-1** | **LDL-2** | **LDL-3** | **HDL** |
| **1** | **G** | **0**  **8**  **16** | 26  26  23 | 20  25  24 | 12  11  8 | 9  15  14 | 34  50  53 | 25  15  11 | 0  0  0 | 47  53  55 |
| **2** | **G** | **0**  **8**  **16** | 36  31  28 | 44  43  29 | 14  12  16 | 17  22  15 | 78  78  66 | 24  25  37 | 1  6  7 | 60  77  65 |
| **3** | **G** | **0**  **8**  **16** | 32  23  25 | 34  36  28 | 14  13  18 | 14  19  23 | 76  88  81 | 37  25  22 | 4  4  3 | 50  53  43 |
| **4** | **B** | **0**  **8**  **16** | 35  28  21 | 51  50  29 | 21  16  12 | 27  23  11 | 74  81  45 | 33  32  48 | 0  0  5 | 59  55  43 |
| **5** | **G** | **0**  **8**  **16** | 24  31  33 | 33  34  34 | 9  11  11 | 24  18  23 | 44  51  65 | 7  10  12 | 0  0  0 | 70  56  59 |
| **7** | **G** | **0**  **8**  **16** | 36  39  21 | 44  36  22 | 19  19  11 | 28  18  23 | 66  59  61 | 30  35  26 | 5  4  2 | 40  47  54 |
| **9** | **G** | **0**  **8**  **16** | 26  32  23 | 36  31  26 | 11  11  12 | 15  16  16 | 41  45  49 | 19  21  13 | 1  2  1 | 43  37  43 |
| **10** | **G** | **0**  **8**  **16** | 32  28  29 | 31  26  32 | 10  13  11 | 26  35  29 | 43  33  52 | 4  3  4 | 0  0  0 | 59  60  65 |
| **11** | **B** | **0**  **8**  **16** | 20  21  18 | 22  17  19 | 7  11  7 | 7  23  15 | 23  22  28 | 13  2  3 | 0  0  0 | 53  50  58 |
| **12** | **B** | **0**  **8**  **16** | 26  32  26 | 14  21  17 | 6  8  6 | 14  19  14 | 46  49  39 | 15  6  3 | 2  0  0 | 61  47  43 |
| **13** | **B** | **0**  **8**  **16** | 20  21  16 | 13  12  13 | 8  6  5 | 5  17  9 | 31  28  36 | 11  3  8 | 0  0  1 | 54  54  47 |
| **14** | **G** | **0**  **8**  **16** | 33  20  20 | 19  17  16 | 7  8  5 | 14  16  9 | 40  36  36 | 6  6  4 | 0  0  0 | 34  31  37 |
| **16** | **B** | **0**  **8**  **16** | 32  30  22 | 17  16  15 | 8  9  7 | 8  16  6 | 29  40  22 | 28  15  27 | 5  0  7 | 32  35  32 |
| **17** | **G** | **0**  **8**  **16** | 38  35  26 | 25  27  26 | 19  14  12 | 14  19  14 | 49  50  42 | 51  26  32 | 6  0  0 | 40  42  49 |
| **18** | **G** | **0**  **8**  **16** | 32  21  22 | 37  25  22 | 9  8  7 | 28  24  12 | 42  38  44 | 9  7  9 | 0  2  3 | 56  54  63 |
| **19** | **G** | **0**  **8**  **16** | 27  24  19 | 34  26  25 | 15  14  11 | 16  16  10 | 51  40  53 | 11  7  14 | 0  0  0 | 55  48  57 |
| **20** | **G** | **0**  **8**  **16** | 34  23  20 | 25  23  28 | 10  11  8 | 11  29  26 | 44  36  56 | 9  2  7 | 0  0  0 | 55  61  79 |
| **21** | **G** | **0**  **8**  **16** | 23  27  24 | 33  28  22 | 10  10  8 | 28  21  19 | 54  57  48 | 6  6  9 | 0  0  0 | 73  76  61 |
| **23** | **G** | **0**  **8**  **16** | 19  15  15 | 21  17  16 | 7  7  6 | 8  20  8 | 47  43  51 | 20  10  16 | 1  2  0 | 57  48  56 |
| **24** | **B** | **0**  **8**  **16** | 27  30  22 | 19  18  15 | 9  11  7 | 10  11  14 | 26  27  36 | 28  21  9 | 2  0  0 | 42  40  49 |
| **25** | **G** | **0**  **8**  **16** | 32  32  30 | 30  28  24 | 10  11  7 | 11  18  15 | 50  56  59 | 23  16  9 | 2  2  0 | 37  35  48 |
| **26** | **B** | **0**  **8**  **16** | 29  53  27 | 29  26  28 | 14  15  11 | 15  14  11 | 59  42  48 | 51  50  41 | 4  9  3 | 49  45  39 |
| **28** | **G** | **0**  **8**  **16** | 38  24  28 | 38  33  24 | 14  7  7 | 31  18  18 | 59  59  64 | 4  6  8 | 0  0  0 | 68  57  71 |
| **29** | **B** | **0**  **8**  **16** | 30  30  27 | 26  32  27 | 10  12  9 | 14  19  13 | 46  62  48 | 17  21  29 | 0  0  1 | 50  41  39 |
| **30** | **B** | **0**  **8**  **16** | 14  18  19 | 17  18  21 | 5  7  4 | 16  20  16 | 38  32  40 | 9  3  3 | 0  0  0 | 57  50  54 |
| Reference range* | | | ≤22 | ≤23 | ≤15 | ≤25 | ≤57 | ≤32 | ≤6 | ≥40 |

*Reference ranges derived from 125 serum samples that met the NCEP ATP III guidelines for desirable lipid status (Quantimetrix Corp. CA, USA)
